# Weekly dose of Iron-Folate Supplementation with Vitamin-C in the workplace can prevent anaemia in women employees

**DOI:** 10.12669/pjms.291.3016

**Published:** 2013

**Authors:** Bobby Joseph, Naveen Ramesh

**Affiliations:** 1 Bobby Joseph, MD, DNB, Professor, Division of Occupational Health Services, Department of Community Health, St. John’s Medical College, Bangalore 560034, India.; 2 Naveen Ramesh, MD, DIH, Assistant Professor, Division of Occupational Health Services, Department of Community Health, St. John’s Medical College, Bangalore 560034, India.

**Keywords:** Intervention, Iron deficiency anaemia, Weekly dose, Workplace

## Abstract

***Objective:*** To assess if a weekly dose of iron and folic acid along with vitamin C, in the workplace would reduce the prevalence of anaemia.

***Methodology:*** A multi-pronged intervention was carried out to reduce the prevalence of anaemia among workers of 7 apparel manufacturing factories using a regime consisting of a supervised single dose of albendazole (400mg) followed by a weekly dose of dried ferrous sulphate (150mg), folic acid (0.5mg) and vitamin C (100mg). Workers were provided information on the causes of anaemia and its prevention. The total duration of the intervention was 16 weeks. Haemoglobin levels of a randomly selected sample of workers were tested before and after the intervention using a computerized non-cyan-meth-haemoglobin method.

**Results:** Of the 10810 workers who were enrolled a sample of 515 workers was randomly selected for the blood investigations. At the end of the intervention (18 weeks after the first blood sample was collected) only 361 out of the 515 who had been enrolled a little more than 16 weeks earlier still remained in the factories and among women 279 out of 385 enrolled were still working in the factories. In the 385 unmatched samples the number of anaemic women had reduced from 141 before the intervention to 79 after - mean haemoglobin increasing from 12.2 to 13.0 (p < 0.001) and in the 279 paired samples prevalence of anaemia had reduced from 105 to 58 - mean haemoglobin increasing from 12.1 to 13.0 (p < 0.001).

**Conclusions:** Our results demonstrated that in resource poor regions, where prevalence of anaemia is high, the workplace may be considered an ideal location to give a weekly supervised dose of iron, folic acid and vitamin C to effectively tackle the problem and probably improve worker efficiency.

## Introduction

 The garment industry in India employs about 3,27,397 individuals working in about 5777 factories producing goods both for the home market and for export.^1^ In Karnataka, about 2,931 garment manufacturing units are functional, of which 2,638 units are situated in Bangalore. The total work force in the state is about 6,50,500 of them 83% are employed in Bangalore.^2^

 Women in the reproductive age group form 80% of the workforce. Many of these workers are the main breadwinners of their families. Reports of projects conducted in this industry have indicated that anaemia is a common problem in this industry.^3^ Anaemia is seventh commonest morbidity that has been documented by factory medical officers – this excludes cases in which it has been seen as a co-morbidity.^4^

 Studies done in the past year in the same group of factories indicate that the prevalence of anaemia is around 62% - of the non-pregnant women 75% were anaemic.^5^ A Non-Governmental Organization with expertise in the health and nutrition of women labour in Karnataka visited each of the factories and has suggested a multi-pronged approach to combat the problem of nutritional anaemia in this industry.^6^

This study was conducted realizing that anaemia is a common problem among women and understanding that industries will be willing to adopt an intervention, if the regimen is essentially low-cost and easily deliverable. It is also important to prove that such an intervention was medically efficacious, with little or no side-effects. Additionally, if productivity improved this model would make “business sense”. The positive impact of this intervention on productivity has been published elsewhere.^7^

## Methodology

 This study was conducted in seven factories belonging to three different companies, all of which manufactured apparel for the export market. At the time of planning the protocol of the study, three months earlier, the seven factories put together employed a total of approximately 10,300 workers. (At the time of commencement of the drug administration there were 10,810 workers). Having listed these workers by name and employee number, a 5% random sample of workers – both males and females – was drawn up, totalling to 515 workers. This sample size was well over and above the sample size that would have been derived had the study presumed that 50% of the workers had been anaemic, using a confidence interval of 95% and a relative deviation of 10%. Fully aware that there is a high attrition rate that plagues the industry in Bangalore City, two back up lists were also drawn up from which, once again, workers could be enrolled randomly to participate in the study.

 Over a period of 10 working days, blood samples of all the 515 randomly selected workers from the factories were collected and analysed using a computerised non-cyan-meth-haemoglobin method. The haemoglobin levels of each of the workers were estimated. 

 Prior to the commencement of the distribution of the drugs all efforts were made to meet with the peer educators in the individual factories, the supervisors and other important stake holders. In this interactive session the employees were told about the intervention, the modality of administration of drugs, the criteria for eligibility for drug consumption, the likely (or unlikely) side effects and other aspects related to the administration of the medications.

 Following this the Medical Officer of the Project and the Research Assistants interviewed each worker on the shop-floor in an attempt to identify if any of them would automatically be disqualified from the process of drug administration. Thus all currently pregnant and those who expressed doubts about the status of their gravidity were noted down. Prior to the commencement of the administration of the drugs the informed consent of the workers was obtained after explaining to them about the process in the vernacular. Thereafter, in the first week of drug administration all the three medications, namely albendazole, iron with folic acid and vitamin C were administered to all consenting individuals. Special care was taken to withhold albendazole from those individuals who expressed doubts about whether they were pregnant or not. The drugs administered were:

* Albendazole 400mg – single dose

* Dried Ferrous Sulphate 150mg and Folic Acid 0.5mg – sixteen weekly doses

* Ascorbic Acid 100mg – also sixteen weekly doses along with ferrous sulphate and folic acid.

At the end of the intervention, as expected, it was found that a large number of individuals had indeed left the factories – only 361 out of the 515 who had been enrolled a little more than 16 weeks earlier still remained in the factories. Of the 385 women workers only 279 remained in the factories. In addition to repeating the haemoglobin levels for these 361 workers, it was decided to replace the 154 workers who had left with an equal number of randomly selected workers taking care to replace each missing worker with one of the same gender and also ensuring that the replacement worker had been working in the factory for the duration of the project i.e. he/she would have had access to the administered drugs.

## Results

 As mentioned earlier, (Table-I) at the time of commencement of drug administration there were a total of 10,810 workers. Of these 9,309 (86.1%) were given the single dose of albendazole – those who did not consume albendazole included mainly those women who expressed even the slightest doubt of being pregnant and a few of those who were not willing to accept the medication. Again 9,263 (85.7%) of the workers consumed 13 or more doses of the iron, folic acid and vitamin C medications. Also shown in Table-I is the pattern of attrition in each of the seven factories, which finally left 361 (70.1%) of the workers remaining in the factory after 16 weeks of the intervention.

**Table-I:** Enrolled workers and medication compliance in the factories

*Factory*	*No. of workers*	*No. of workers who consumed Albendazole*	*No. of workers who consumed > 12 doses*	*No. of workers enrolled for study*	*No. remaining at end of 16 weeks*
Factory A	1750	1628	1605	113	81
Factory B	2370	2068	2128	121	66
Factory C	1243	1169	1154	63	53
Factory D	1233	878	866	44	33
Factory E	1098	800	846	32	28
Factory F	1346	1243	1204	55	43
Factory G	1770	1523	1460	87	57
Total	10810	9309	9263	515	361

***The 515 un-matched sample (*** Table-II ***):*** The analysis of data for the 515 un-matched samples showed that the number of anaemic individuals had dropped by 69 (46.6%). The mean haemoglobin for this sample had also increased by 0.8g/dL. Both these differences were statistically significant.

***The 361 matched sample (*** Table-III ***):*** It could be argued that 154 workers, although who replaced those who had left in the un-matched sample (although randomly selected) were inherently of better haemoglobin status. Hence, it was decided to run the statistical tests on the matched samples of 361 workers. Here too it was found that the number of anaemic individuals had dropped by 49 (45.8%) and that the mean haemoglobin for this matched sample had increased by 0.89g/dL. Again, these differences were found to be statistically significant.

***Gender of employees (*** Table-IV ***):*** Given the fact that more than 25% of the workers enrolled for the study were males, it was also possible that males benefited more from the intervention than the females. While the χ^2^ test did not reveal any significant difference in the anaemic status of males, the reduction in the number of anaemic females was found to be significant. However, the *t* tests revealed that there were significant changes in both groups, both among the matched samples and the un-matched samples.

## Discussion

***The role of single-dose Albendazole:*** Studies^8^ have demonstrated that peak age-specific prevalence of infection with intestinal helminths occurs in adult life, but only a small proportion of those infected ever become symptomatic. Papers published following meta-analysis of recent studies^9^^,^^10^ have suggested that for the control of transmission of hookworm infection, periodic chemotherapy should be implemented in the context of on-going improvement of sanitation and promotion of health education. These elements should be integrated into the prevailing system of primary health care and must be based on multi-sectoral collaboration to ensure sustainability of control programs. These studies have demonstrated a positive impact of anti-helminthic treatment on haemoglobin levels, with best results obtained in settings where iron intakes were also increased.

 A report from Sri Lanka^11^ has indicated that in areas where hookworm is a major problem and albendazole is used regularly along with iron supplements, mass chemotherapy appears to give maximal returns in terms of improved health. Single-dose, broad-spectrum anti-helminthic drugs given in low doses at prolonged intervals can maintain worm burdens below pathogenic levels for almost all of the major human helminth parasites – a longitudinal study done in Sierra Leone to measure the effect of a single dose albendazole and daily iron-folate supplement also showed an increase in haemoglobin level among pregnant women.^12^ Warren^13^ has suggested that albendazole or one of the other benzimidazoles can be used for hookworm, ascaris and trichuris. Treatment of soil transmitted helminthic infection with a single dose of any one of the four anti-helminthic of the WHO list of essential drug is found to be cost effective.^14^

**Table-II:** Characteristics of the un-matched sample of males and females (n = 515).

*Parameter*	*Pre-intervention*	*Post-intervention*	*Remarks*
Anaemic	148	79	χ^2^ = 26.90 p < 0.001
Mean haemoglobin	12.93	13.73	t = 6.73 p < 0.001
Standard deviation	2.03	1.93	
Variance	4.14	3.71	
Range	3.0-17.8	6.3-19.2	
Inter-quartile range	11.8-14.2	12.7-14.9	
Median	12.9	13.7	
Mode	12.6	14.0	

**Table-III:** Characteristics of the matched sample males and females (n = 361).

*Parameter*	*Pre-intervention*	*Post-intervention*	*Remarks*
Anaemic	107	58	χ^2^ = 17.33 p < 0.001
Mean haemoglobin	12.83	13.72	t = 19.53 p < 0.001
Standard deviation	2.00	1.96	
Variance	3.99	3.85	
Range	6.4-17.7	7.8-19.2	
Inter-quartile range	11.8-14.1	12.7-15.0	
Median	12.8	13.7	
Mode	12.6	13.1	

 This study draws on the results shown in the above papers. However, it must be admitted that worm loads were not estimated either before or after the intervention, hence it is not possible to make conclusions on effect of albendazole on this sample of workers.

***The role of weekly dose of Iron/Folic Acid/Vitamin C:*** Concluding an experiment in the workplace aimed at enhancing haemoglobin levels using different methods, one study^15^ concludes that enhancing the haemoglobin levels of young working women makes good economic sense. Such a strategy brings about good labour relations apart from the established rewards of greater productivity, better reproductive health, better cognition and an all-round better working atmosphere.

 While acknowledging that the most promising approach is dietary improvement by iron fortification of common staples^16^^,^^17^ or even by improving the quality of food consumed, it is difficult to adopt this approach in an industry where the one meal provided is on an optional basis – a number of women bring home cooked meals for the luncheon break.

 A consultative group of scientists have suggested that in situations where there is evidence of anaemia as a public health problem, oral iron supplementation of adolescents and women of child bearing age is recommended.^18^ Efficacy study done among school children, adolescents and pregnant mothers has shown weekly or twice weekly iron supplement is as effective as daily supplementation.^19^^-^^21^

**Table-IV:** Results of the intervention in females alone

*Parameter*	*385 unmatched females*		*279 females with paired samples*	
	*Pre*	*Post*	*Pre*	*Post*
No. Anaemic				
M Hb< 13.0, F Hb < 12.0	141	74	105	58
Mean Haemoglobin	12.16	13.00	12.12	12.98
Standard Deviation	1.62	1.45	1.59	1.49
Variance	2.62	2.10	2.54	2.21
Range	3.0-15.4	6.3-16.1	6.4-13.2	7.8-16.1
Inter-quartile range	11.4-13.2	12.3-14.0	11.3-13.2	12.2-14.0
Median	12.5	13.2	12.4	13.2
Mode	12.8	13.1	13.1	13.1
t	7.64, p < 0.001		16.83, p < 0.001	

 Trials have shown that weekly iron/folic acid supplementation can be efficacious in controlling anaemia if supplements are taken regularly.^22^ The international expert group mentioned above^18^ affirms that in some situations weekly supplementation is efficacious as long as compliance is achieved. They state that the lower cost of the dosage, lesser frequency of side effects and the possibility of its promotion as a weekly event in communities, supported by communication activities make it attractive if it can be shown to be effective in practical programs. Quoting from a number of studies, a recent review^17^ states that the strategy of long-term weekly iron-folate supplementation is a practical, safe, effective and inexpensive method for improving iron nutrition in adolescent girls. Again quoting from personal communications in a report to Micronutrient Initiative and UNICEF, Gillespie states that weekly supplementation of iron to female factory workers is already part of national policy in Indonesia.^23^

 Vitamin C is a strong promoter of iron absorption from the diet.^24^ The addition of 50mg of Vitamin C per day improved iron status in children over a period of 8 weeks.^25^ Prophylactic administration of iron along with antioxidants like vitamins E and C or foods rich in these vitamins is one strategy to combat the role of excess iron in causing intestinal oxidative stress.^16^

 A study^26^ from Indonesia indicates that reporting of side effects such as nausea, vomiting, diarrhoea and sleepiness were least when a combination of iron 60mg, vitamin C 60mg, folate 500mg and Retinol 6000 micrograms was given when compared to a daily regimen consisting of similar quantities of the first three ingredients and 750micrograms of retinol or a high dose of iron (120mg). These medications were administered for period of 12 weeks. The Sierra Leone study^12^ also demonstrated that single dose of 400mg albendazole and daily dose of 36mg of iron and 0.5mg folate during pregnancy increased the haemoglobin concentration by 6.6 g/L between baseline and third semester.

 Our experimental study has revealed that even a small dose of iron given on a weekly basis can significantly reduce the number of anaemic individuals in a given population. Moreover, there is a significant improvement in the mean haemoglobin levels. It was heartening to note that this finding did not confine itself to the already robust males but had also had a significant difference in the females in whom the problem of anaemia is more acute.

***Side Effects:*** Like mebendazole, albendazole produces few side effects^27^ when used for short term therapy of gastro-intestinal helminthiasis even in patients with heavy worm burdens. Transient abdominal pain, diarrhoea, nausea, dizziness and headache may occur on occasions. In the case of iron and folic acid, side effects are fewer in those receiving weekly doses as compared to those receiving daily doses. In the latter these include heartburn, nausea, upper gastric discomfort, constipation and diarrhoea.

 None of the workers approached the Medical Officer (MO) or the Research Assistants (RA) in the week following the administration of albendazole with complaints of side-effects. On further probing, a few workers said they had abdominal pain and itching – none of which warranted a visit to a health facility either within or outside the factory premises. Similarly, very few workers (less than 1%) refused to take any iron and folic acid tablets whatsoever – the common reasons for these were that they were already taking some prophylaxis prescribed by their own general practitioners or because they were “already healthy”. Similarly a few who dropped out of the study did so because they suffered from gastritis, itching, loose motions or tiredness. However, none of these workers self-reported these symptoms to the MO or RA.

 It is our inference, therefore, that albendazole, iron, folic acid and vitamin C given in mass campaigns in a frequency similar to this is cost effective and with little or no side effects or issues related to compliance.

## Conclusions

 This study has revealed that a small dose of iron and folic acid, given in combination with vitamin C, at weekly intervals is sufficient to improve the haemoglobin levels in women workers on the shop-floor, while also reducing the number anaemic women. Given the possibility that worm infestations are very common in our population, there is a role for administering anti-helminthics at regular intervals. The low cost of the drugs, the relative absence of side effects and the ease of administration of the medications on the shop-floor indicate that the intervention is only of benefit to the factory.

## Notes

***Note: ***A part of this study was present at the 57^th^ Conference of the Indian Association of Occupational Health, Chennai in February 2006.

## Notes

***Source of support:*** International Youth Foundation through Global Alliance for Workers and Communities grant.
